# A psychophysical and neuroimaging analysis of genital hedonic sensation in men

**DOI:** 10.1038/s41598-022-14020-4

**Published:** 2022-06-17

**Authors:** Gerben B. Ruesink, Francis P. McGlone, Håkan Olausson, Camilla de Jong, Jan-Bernard Marsman, Remco J. Renken, Janniko R. Georgiadis

**Affiliations:** 1 Department of Biomedical Sciences of Cells & Systems, University of Groningen, University Medical Center Groningen, Groningen, The Netherlands; 2 Research Centre for Brain & Behaviour, School of Natural Sciences & Psychology, Liverpool John Moores University, Liverpool, UK; 3 Department of Biomedical and Clinical Sciences, Center for Social and Affective Neuroscience, Linköping University, Linköping, Sweden; 4 Netherlands Institute for Mental Health and Addiction (Trimbos Institute), Utrecht, The Netherlands

**Keywords:** Reward, Sexual behaviour, Somatosensory system

## Abstract

Current understanding of human genital-brain interactions relates primarily to neuroendocrine and autonomic control, whereas interactions during sexual stimulation remain largely unexplored. Here we present a systematic approach towards identifying how the human brain encodes sensory genital information. Using a validated affective touch paradigm and functional magnetic resonance imaging, we found that hedonic responses to discriminatory versus affective tactile stimulation were distinctly different for both penile shaft and forearm. This suggests that, as with other body sites, genital skin contains small diameter mechanoreceptive nerve fibres that signal pleasant touch. In the brain, secondary somatosensory cortex (S2) distinguished between affective and discriminative touch for the penile shaft, but not for the forearm. Frenulum stimulation induced the greatest reports of subjective pleasure and led to the greatest deactivation of the default-mode network. This study represents a first pass at investigating, in humans, the relationship between innervation of genital surfaces, hedonic feelings, and brain mechanisms, in a systematic way.

## Introduction

Of all human skin sites, the external genitalia are considered the areas with the highest potential for tactile pleasure^1^. This observation may be seen as an expression of the essential function of genitalia in procreation, more specifically the strong tie between sexual activity and reward^2^. However, very little is known about the way the central nervous system converts genital afferent somatosensory information into a pleasurable experience. There are at least two reasons for this: our limited understanding of the precise stimulatory response properties of peripheral nerves innervating the various genital surfaces, and the limited data available on the affective aspect of human genital tactile stimulation. Thus, we are in a sense oblivious to an important part of our affective and emotional lives.

Focusing on the sense of touch (and disregarding thermosensation and chemosensation) there is general agreement that this sense can be subdivided into discriminative and affective components^3,4^. Affective touch is phylogenetically older, is conveyed by slowly conducting unmyelinated fibers, has relatively poor spatial localization potential, is more likely to cause an emotional response, and has been argued to play a role in bodily feelings and social wellbeing^3^. In the brain, affective touch is primarily processed in the posterior insula (pINS), whereas discriminative aspects of touch are mainly processed in the primary somatosensory cortex (S1)^5–8^. Given the high pleasure potential of genital touch^1^, it would make sense that the affective component of the touch system in the brain plays a major role in processing genital touch.

The affective properties of touch are understood to be first encoded by a special class of gentle touch sensitive C-fibres in the skin of the body called C-tactile afferents (CT). Physiologically, these C-tactile (CT) afferents respond optimally to innocuous, dynamic (moving) stimulation with low force and at a specific velocity corresponding to a caress^9–11^. Psychologically, this type of stimulation is pleasurable, and in fact more pleasurable than faster and slower stimulations^12,13^. Psychophysical paradigms can reliably and selectively target CT-fibres and have been shown to produce highly reproducible pleasure responses^14^. However, it is currently unknown which fibre types are essential for signalling pleasurable genital sensations. Genital skin, in particular the glans, has an abundance of small-diameter fibers^15,16^, suggesting that the type of psychophysical paradigm as used in CT research is adequate to study genital affective touch in a controlled fashion.

In the brain, CT-targeted stimulation appears to be primarily processed by the pINS^5–8^, a paralimbic somatosensory area that is considered important for interoception, i.e. monitoring the body’s internal state. There is some evidence that the pINS has a somatotopic map for C-fiber afferent stimulation^6^, including thermal and noxious information^17–20^. The pINS is well-connected to agranular limbic cortices, such as the anterior insula (aINS) and dorsal anterior cingulate cortex (dACC), which drive motivation and motivated behaviour, and to the secondary somatosensory cortex (S2)^21–23^. It is therefore not surprising that these areas are commonly found to be involved in CT processing as well^5–8^.

Interestingly, there seems to be much overlap with the central processing of sexual genital stimulation in this regard. All parts of the insula, as well as the dACC and S2, have been related to the human sexual response, with a bias towards paradigms that evoked penile erection or that involved genital touch stimulation^2,24,25^. Such studies involve complex situations where subjects are sexually aroused either because of sexually intended tactile stimulation provided by a partner, or because of prolonged visual sexual stimulation causing genital responses. These complicated experimental setups make it difficult to relate brain activity patterns to specific aspects of genital innervation and sensation, such as different types of mechanosensory input (pressure, dynamic touch) and their psychological consequences. Specifically, insula and S2 responses have been related to penile tumescence, penile touch, sustained psychological sexual arousal, and combinations of these^26–29^. Thus, some brain areas seem to express a tendency to activate both during an affective state involving genital stimulation (as seen during sexual activity) and during non-genital, non-sexual affective touch. This makes these areas prime candidates for establishing a link between human genital skin and positive affective tone.

A striking discrepancy in the central processing of general affective touch and genital stimulation lies in the presumed role and contribution of S1. This area has been attributed at most a minor role in the processing of CT afferent information^5,6,8^ (but see^30^); yet, in human studies of (sexual) genital afferent processing it represents perhaps the most important focus, mainly because of its role in scrutinizing the classic concept of the somatosensory ‘homunculus’^31–35^. Efforts to localize the primary genital somatosensory representation have led to debate^35,36^, but unfortunately this has not resulted in breakthroughs regarding the functional pathways in the brain for genital afferent signals relevant to e.g. sexual behaviour.

It is thus clear that the lack of knowledge on peripheral innervation of genital surfaces, together with the focus on S1 mapping in genital touch human neuroimaging studies, has precluded analysis of subjective experience in relation to affective genital afferent stimulation. Yet, only thorough and systematic analysis of the relation between genital innervation, characteristics of the genital stimulation and corresponding genital sensations, will reveal how the genitalia contribute to our affective lives. Moreover, such an analysis can provide an important first step towards informed and systematic investigations of genital stimulation relevant to sexual function and arousal. In the present study, we investigated the affective properties of penile touch on both a subjective and neural level, using the penile skin as a model of genital surfaces in general, and asserting that genital touch may have a prominent affective component. We assessed subjective pleasure ratings and fMRI BOLD responses reflecting brain activity during brushing stimulation of the forearm, penile shaft, and penile frenulum. Specifically, we hypothesized that stimulation of the penile hairy skin targeted at affective touch fibres (CT-targeted) would result in both higher pleasure ratings and stronger activity in the pINS and S2, compared to stimulation aimed at other discriminatory fibre types (non-CT-targeted).

## Results

### Psychophysical results

**Figure 1:**
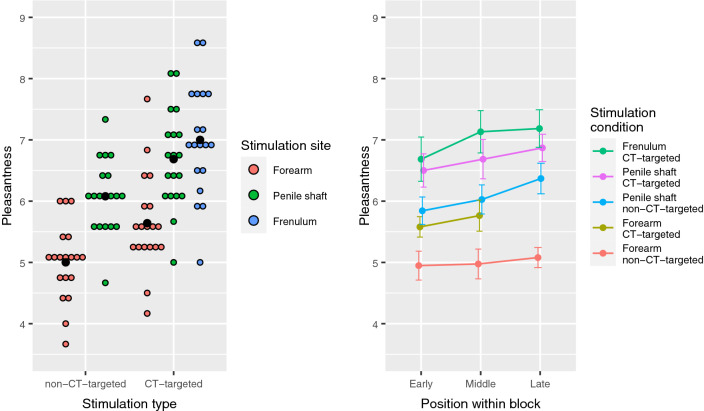
Overview of mean pleasantness scores (N = 19) per dynamic touch condition. Ratings were given on a scale from 0 (‘very unpleasant’) to 10 (‘very pleasant’). *Left panel:* The scores show a linear pattern where penile shaft stimulation was preferred over forearm stimulation (*p* < 0.0001) and CT-targeted stimulation was preferred over non-CT-targeted stimulation (*p* < 0.0001). There was no significant interaction between stimulus type and stimulus site. Coloured dots indicate individual participant means and black dots indicate the group mean. *Right panel:* Pleasure ratings for all stimulation types were also influenced by the position within a stimulation block. A later (vs. earlier) position within a stimulation block enhanced perceived pleasure in a linear way (*p* < 0.001). In this panel, data points are averaged over subjects and sessions, and error bars indicate 95% confidence intervals. Figures were created using *ggplot2 (v. 3.3.2; *https://ggplot2.tidyverse.org/) within RStudio (v. 1.4.1106; https://www.rstudio.com/).

The subjective pleasantness ratings show a clear pattern (see Fig. 1; left panel), where penile dynamic touch was perceived as more pleasurable than forearm dynamic touch and CT-targeted stimulation was more pleasant than non-CT-targeted stimulation. Three subjects reported having had a (partial) erection at some time during the experiment. We created two linear-mixed effects models (LMMs) to statistically assess these patterns. In the first LMM, we excluded the frenulum condition, so we could test for a potential Velocity*Location interaction. The LMM confirmed the observations that penile shaft stimulation was preferred over forearm stimulation (ß = 1.08, T [432] = 11.53, *p* < 0.0001), and that CT-targeted stimulation was preferred over non-CT-targeted stimulation (ß = 0.76, T [432] = 7.71, *p* < 0.0001), but revealed no significant Velocity*Location interaction. While accounting for any potential effects of time or stimulation order, we found that the position of each stimulus within a stimulation block (relative to the other stimuli) affected the perceived pleasure in a linear way (Fig. 1; right panel), where a late (vs. early) position within a block enhanced perceived pleasure (ß = 0.35, T [432] = 3.90, *p* < 0.001). In the first LMM, between-subject differences in intercept accounted for 35.0% of variance, and inclusion of Subject as random factor significantly improved the model fit (LRT [1] = 179.82, *p* < 0.0001).

The second LMM included all stimulation conditions, with the frenulum condition as intercept, so we could test how frenulum stimulation compared to the other stimuli. While accounting for effects of position within block (see previous paragraph), we found that frenulum stimulation was reported as being significantly more pleasant than all other stimulation types, with the smallest difference compared to CT-targeted stimulation of the penile shaft (ß = − 0.32, T [545] = − 3.01, *p* < 0.005) and the largest difference with non-CT-targeted forearm stimulation (ß = − 2.00, T [545] = − 19.06, *p* < 0.0001). In this model, between-subject differences in intercept accounted for 29.6% of variance, and here as well inclusion of Subject as random factor significantly improved the model fit (LRT [1] = 161.39, *p* < 0.0001).

### fMRI results

**Figure 2:**
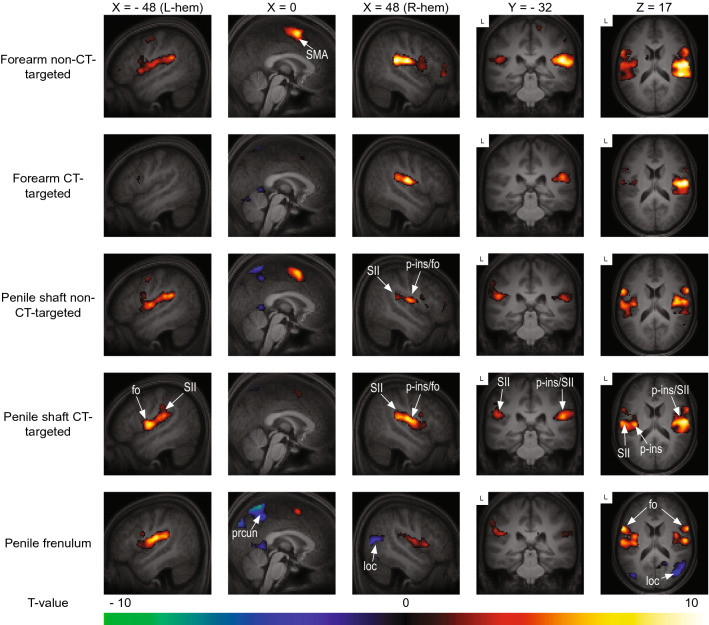
Group level results of the whole-brain analysis of each dynamic touch condition versus baseline (fixation). Red indicates activation relative to baseline, blue indicates deactivation relative to baseline. The template was created by averaging the structural T1-scans of the 19 participants. Results are presented at a threshold of *p* < 0.05 FWE-corrected. *Abbreviations: frontal operculum (fo), lateral occipital cortex (loc), posterior insula (p-ins), precuneus (prcun), supplementary motor area (SMA), secondary somatosensory cortex (SII).* FMRI sections were created using *FSLeyes* (v. 1.3.3; https://fsl.fmrib.ox.ac.uk/fsl/fslwiki/FSLeyes). Final figure was created using *Adobe InDesign 2022 for Mac* (v. 17.2; https://www.adobe.com/products/indesign.html).

Both tactile stimulation of forearm and penis evoked significant activation of S2, pINS supplementary motor cortex (SMA), lateral precentral gyrus, and parts of the superior temporal gyrus (*p* < 0.05 family wise error [FWE] corrected). S2 is located in the upper bank of the lateral sulcus and includes the most lateral aspect of the postcentral gyrus and the central and parietal operculum^37^. As can be seen in Fig. 2 and Table 1 there is remarkable overlap in brain response between the various conditions.

**Table 1:** Activated regions, MNI-coordinates, and T-statistics for each of the dynamic touch conditions versus baseline.

Condition	Region	Cluster size	T-value	x	y	z
*Activations*						
Penile shaft non-CT-targeted	L Central operculum (S2), extending to lateral postcentral gyrus (S2) and precentral gyrus	3363	10.54	− 60	6	24
			8.84	− 60	− 18	22
			8.61	− 44	− 6	8
	R Central operculum (S2), extending to lateral precentral gyrus	2738	9.08	58	− 16	20
			8.81	42	− 12	14
			8	60	8	14
	L & R Supplementary Motor Cortex	1281	8.7	− 4	4	58
	L Anterior insula	131	6.48	− 30	22	6
Penile shaft CT-targeted	R Central operculum (S2), extending to supramarginal gyrus	3430	10.44	54	− 16	20
			9.61	54	− 4	8
			9.12	58	− 20	30
	L Central operculum (S2), extending to lateral precentral gyrus	3428	8.96	− 58	8	24
			8.68	− 48	− 6	8
			8.66	− 60	− 18	22
	L & R Supplementary Motor Cortex	160	5.78	4	8	58
	R Anterior insula	77	6.15	32	22	0
	L Anterior insula	31	5.73	− 32	20	4
Penile frenulum	L Central operculum (S2), extending to lateral postcentral gyrus (S2) and precentral gyrus	3912	11.22	− 58	8	24
			9.86	− 60	− 18	22
			9.55	− 44	− 6	8
	R Central operculum(S2), extending to lateral precentral gyrus	2410	9.53	56	− 16	20
			8.08	60	8	14
			7.97	54	8	6
Forearm non-CT-targeted	R Central operculum (S2), extending to planum temporale (STG)	3993	10.17	58	− 16	18
			9.95	50	− 34	18
			9.81	48	− 16	18
	L Central operculum (S2), extending to lateral precentral gyrus and planum temporale (STG)	3514	8.76	− 60	6	24
			7.37	− 48	− 40	22
			7.26	− 40	− 16	14
	L & R Supplementary Motor Cortex, extending to precentral gyrus and paracingulate gyrus	1378	9.4	− 4	6	58
			6.29	10	− 26	68
			5.8	− 4	22	44
	R Frontal pole	295	6.34	48	40	− 6
			6.29	38	42	− 4
			5.97	44	40	6
	R Supramarginal gyrus (posterior division)	84	6.01	56	− 44	50
			5.11	54	− 34	48
	R Planum polare (STG)	44	5.67	42	− 10	− 12
			5.17	38	0	− 16
	L Caudate nucleus	36	0.331	− 14	− 2	16
			0.58	− 12	8	12
Forearm CT-targeted	R Central operculum (S2), extending to lateral postcentral gyrus (S2)	2101	10.16	56	− 12	16
			9.66	46	− 16	16
			8.58	60	− 14	38
	L Posterior insula	118	6.1	− 38	− 12	14
	L Lateral postcentral gyrus (S2)	146	6	− 58	− 12	18
			5.42	− 56	− 16	38
	L Supplementary Motor Cortex, extending to paracingulate gyrus	121	5.64	− 4	6	58
			5.47	− 4	10	50
			5.33	− 4	22	42
	L Inferior frontal gyrus	136	6.83	− 56	10	22
			4.94	− 46	10	10
	R Lateral precentral gyrus, extending to inferior frontal gyrus	77	5.9	60	10	16
			5.17	54	16	14
	L Planum temporale (STG)	33	5.93	− 58	− 16	0
*Deactivations*						
Penile shaft non-CT-targeted	L & R Precuneus	567	7.23	2	− 52	66
			6.79	2	− 66	60
			6.64	6	− 48	48
	L & R Posterior cingulate & lingual gyrus	335	6.91	8	− 46	0
			6.18	− 2	− 50	4
	R Cerebellum, extending to temporal occipital fusiform cortex	241	6.31	34	− 46	− 24
			5.89	36	− 54	− 20
			5.45	28	− 52	− 20
	L Cuneus	56	6.1	− 4	− 84	38
	L Cerebellum	85	5.76	− 34	− 52	− 24
	R Occipital fusiform & Lateral occipital cortex	65	5.67	36	− 78	− 18
	L Occipital fusiform & Lateral occipital cortex	29	5.42	− 40	− 74	− 16
	R Lateral occipital cortex (superior)	21	5.32	34	− 76	28
Penile shaft CT-targeted	R Lingual gyrus	47	5.73	6	− 48	0
	L Posterior cingulate gyrus	28	5.5	− 8	− 46	0
	L & R Precuneus	39	5.37	2	− 58	62
Penile frenulum	L & R Precuneus	5917	9.27	6	− 62	62
			8.93	2	− 52	64
			8.67	6	− 48	46
	L & R Posterior cingulate & lingual gyrus	1418	9.02	8	− 46	0
			8.06	− 10	− 48	0
			7.46	12	− 54	6
	L Temporal occipital fusiform cortex & lateral occipital cortex (inferior)	491	6.7	− 38	− 64	− 16
			6.34	− 40	− 76	− 14
			5.88	− 34	− 48	− 22
Forearm non-CT-targeted	N.S	N.S	n.s	n.s	n.s	n.s
Forearm CT-targeted	R Cerebellum	819	7.3	24	− 52	− 26
			6.2	18	− 60	− 18
			6.03	4	− 68	− 14
	L & R Cerebellum	127	6.25	2	− 52	0
	L Cerebellum, extending to occipital fusiform cortex	263	6.13	− 38	− 60	− 22
			5.82	− 30	− 54	− 24
			5.75	− 42	− 70	− 18
	L & R Precuneus	127	5.22	6	− 60	62
			4.93	0	− 52	66
			4.93	14	− 60	66

Only activity with a peak-level *p* value < 0.05 (FWE-corrected) and minimal cluster size of (k = 20) is reported. Regions were defined with the Harvard–Oxford cortical atlas. Abbreviations: R, right hemisphere; L, left hemisphere.

Still, close visual inspection yields important differences between forearm and penile conditions. For the forearm, activation clusters in S2 are present bilaterally but have a strong emphasis on the right S2, which is contralateral to the brushing stimulation. For the penile shaft and frenulum, the differences between left and right S2 activity were smaller and more variable. Penile shaft stimulation also evoked significant activity in the ipsi- (both slow and fast stroking) and contralateral (only slow stroking) aINS, which was not the case for frenulum and forearm stimulation.

For the penile stimulation conditions, there was bilateral deactivation of the precuneus, with the strongest effect for frenulum stimulation. A similar but smaller effect was also found for the forearm, but only for CT-targeted stimulation. Additionally, there was deactivation of cerebellum (shaft CT-targeted and forearm CT-targeted), temporal occipital fusiform cortex (shaft CT-targeted and frenulum), lateral occipital cortex (shaft non-CT-targeted and frenulum), lingual gyrus (shaft CT-targeted and frenulum) and posterior cingulate cortex (shaft both CT- and non-CT-targeted and frenulum).

**Figure 3:**
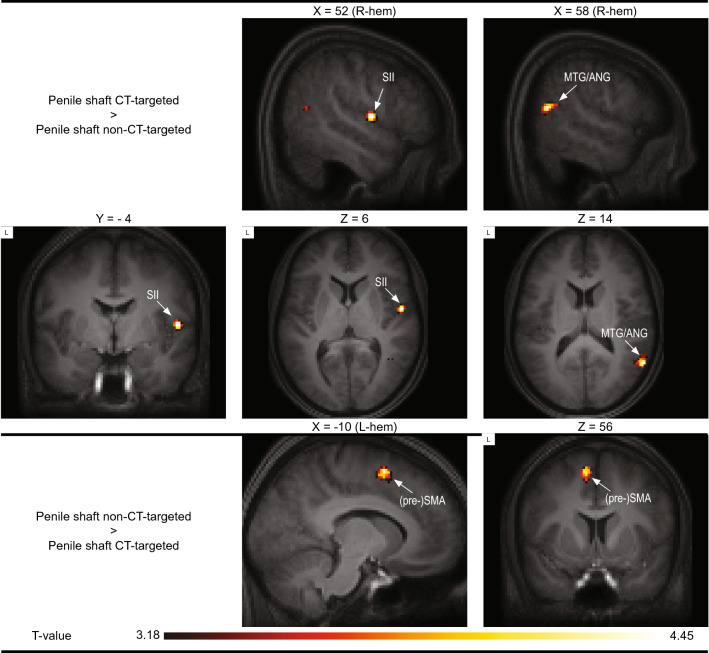
Group level results of the comparison contrast between CT-targeted touch and non-CT-targeted touch of the penile shaft. The template was created by averaging the structural T1-scans of the 19 participants. Results are presented at an uncorrected threshold of *p* < 0.001. *Abbreviations: angular gyrus (ANG), middle temporal gyrus (MTG), (pre-)supplementary motor area (pre-SMA), secondary somatosensory cortex (SII).* FMRI sections were created using *FSLeyes* (v. 1.3.3; https://fsl.fmrib.ox.ac.uk/fsl/fslwiki/FSLeyes). Final figure was created using *Adobe InDesign 2022 for Mac* (v. 17.2; https://www.adobe.com/products/indesign.html).

**Figure 4:**
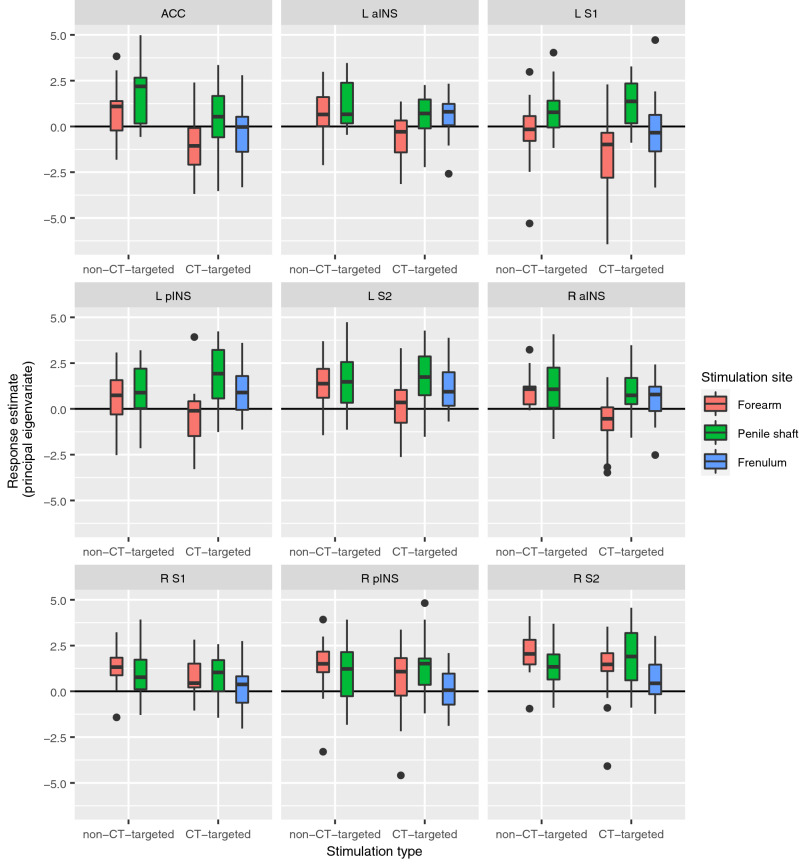
Boxplots of response estimates (principal eigenvariate) for each dynamic stimulation condition for core somatosensory areas. *Abbreviations: primary somatosensory cortex (S1), secondary somatosensory cortex (S2), (bilateral) anterior cingulate cortex (ACC), posterior insula (pINS), anterior insula (aINS).* Figure was created using *ggplot2* (v. 3.3.2; https://ggplot2.tidyverse.org/) within *RStudio* (v. 1.4.1106; https://www.rstudio.com/)*.*

The results of the direct statistical comparison between the various conditions can be seen in Table 2, with some effects highlighted in Fig. 3. Furthermore, in Fig. 4 we present response estimates of regions most reported in affective touch research (S1, S2, ACC, pINS, aINS, all bilateral) as to provide information about the behavior of these core somatosensory areas over the experiment. As can be inferred from Fig. 4, responses in these areas showed less consistency than anticipated. CT-targeted stimulation of the forearm, which has regularly been reported to activate the posterior insula in affective touch studies, yielded quite variable responses in the posterior insula in the present study. As a general picture, there was a clear tendency for core somatosensory areas to respond stronger to non-CT-targeted forearm stimulation relative to CT-targeted stimulation of the same body part; this effect seemed far less clear, and sometimes reversed (CT-targeted stimulation resulting in stronger activation), for the penile shaft. Finally, in these core somatosensory areas responses to frenulum stimulation seemed generally lower than responses to penile shaft stimulation.

**Table 2:** Results of the comparison between CT-targeted versus non-CT-targeted stimulation of the penile shaft, CT-targeted and non-CT-targeted penile shaft versus frenulum stimulation and CT-targeted versus non-CT-targeted forearm stimulation.

Condition	Region	Cluster size	T-value	x	y	z	FWE-corrected *p* value at peak-level
Penile shaft CT-targeted > Penile shaft non-CT-targeted	R Central operculum (S2)	54	3.96	52	− 4	6	< 0.1 *
	R Middle temporal gyrus & angular gyrus	104	3.92	58	− 58	14	n.s
			3.42	42	− 54	8	
			3.34	50	− 56	12	
Penile shaft non-CT-targeted > Penile shaft CT-targeted	L Pre-Supplementary Motor Area	132	4.45	− 8	6	56	n.s
Penile frenulum > Penile shaft CT-targeted	No suprathreshold clusters						
Penile shaft CT-targeted > Penile frenulum	R Lateral occipital cortex (inferior), extending to middle temporal gyrus (MTG)	4017	5.96	50	− 60	12	< 0.005
			5.87	46	− 68	10	
			5.77	44	− 56	8	
	R Temporal occipital fusiform cortex	387	5.15	38	− 46	− 18	< 0.05
			3.93	32	− 34	− 18	
			3.71	26	− 42	− 16	
	R Posterior insula	76	4.47	34	− 18	0	< 0.05 *
	R Precuneus	263	4.36	16	− 60	12	n.s
			3.56	16	− 48	− 2	
	L Lateral occipital cortex (inferior)	187	4.19	− 44	− 74	12	n.s
	R Central operculum (S2), extending to planum temporale (STG)	292	4.06	64	− 10	6	n.s
			3.9	54	− 4	6	
			3.49	52	− 18	10	
	L Planum temporale and Hesch's gyrus	75	4.04	− 48	− 26	4	n.s
	R Superior temporal gyrus (STG)	57	3.92	58	− 6	− 12	n.s
			3.42	46	− 10	− 14	
	R Lingual gyrus	68	3.74	22	− 60	− 10	n.s
	R Superior parietal lobule (SPL)	115	3.7	28	− 40	52	n.s
			3.61	32	− 54	56	
			3.28	38	− 42	62	
	L Anterior insula	31	3.59	− 34	8	− 12	n.s
Penile frenulum > Penile shaft non-CT-targeted	No suprathreshold clusters						
Penile shaft non-CT-targeted > Penile frenulum	No suprathreshold clusters						
Forearm CT-targeted > Forearm non-CT-targeted	No suprathreshold clusters						
Forearm non-CT-targeted > Forearm CT-targeted	L Caudate nucleus	4695	5.55	− 16	− 6	18	< 0.005
			5.23	− 28	4	− 12	
			5.1	− 26	8	− 4	
	R Cerebellum	3993	5.43	18	− 60	− 22	< 0.005
			5.19	− 24	− 62	− 24	
			4.62	30	− 60	− 28	
	L + R Pre-Supplementary Motor Area, extending to anterior cingulate cortex	1197	5.11	− 6	4	60	< 0.01
			4.84	− 2	− 8	70	
			4.71	− 8	− 14	70	
	R Frontal pole	1584	4.78	46	46	− 4	< 0.05
			4.63	44	26	− 8	
			4.57	38	32	18	
	L Supramarginal gyrus (posterior division)	510	4.68	− 52	− 46	20	< 0.05
			4.35	− 62	− 40	16	
	L Precentral gyrus	145	4.47	− 44	− 10	56	< 0.1
			3.55	− 34	− 12	64	
			3.48	− 50	− 6	48	
	L Hippocampus	149	4.38	− 20	− 22	− 8	n.s
	R Supramarginal gyrus (posterior division)	529	4.36	52	− 42	48	n.s
			4.29	48	− 36	42	
			3.51	56	− 44	38	
	L + R Anterior cingulate cortex	44	3.81	0	− 6	32	< 0.05 *
	R Middle temporal gyrus (temporooccipital part)	28	3.47	60	− 54	2	n.s

Only activity with a peak-level uncorrected *p* value < 0.001 and minimal cluster size of (k = 20) is reported. Regions were defined with the Harvard–Oxford Cortical Atlas. * Indicates that the cluster was only significant at an FWE-corrected threshold after applying small-volume correction (SVC). The SVC was applied for a mask containing all regions-of-interest (ROI’s). See Methods-section for more detail on ROI definition. Abbreviations: n.s., non-significant; R, right hemisphere; L, left hemisphere.

The direct comparison of most interest to the present research question, CT vs. non-CT-targeted stimulation of the penile shaft, yielded activation in right S2, i.e. contralateral to the left-sided penile shaft stimulation (Fig. 3).

Compared to the frenulum, CT-targeted stimulation of the penile shaft evoked significant responses in the right posterior insula (contralateral to penile shaft stimulation), and in areas of the right occipitotemporal cortex. As Figs. 2 and 4 also clearly show, these effects largely resulted from increased deactivation during frenulum touch. Interestingly, many of these areas that deactivate during frenulum stimulation are part of the posterior part of the brain’s default mode network.

CT-targeted penile shaft stimulation also evoked increased contralateral S2 activation, when compared to frenulum stimulation*,* whereas non-CT-targeted stimulation resulted in increased activity in the ipsilateral (pre)SMA. Still, neither of these effects survived FWE-correction. Inverse contrasts were also assessed but yielded no significant results, indicating that frenulum stimulation did not activate any brain areas more strongly than shaft stimulation did.

Identifying the effects of CT-targeted stimulation of the forearm was not the primary objective of this study. Still, it is noteworthy that we could only find significant contralateral S1 activation for the forearm (compared to baseline) when using an uncorrected threshold and found no differences in this area between CT and non-CT-targeted stimulation. Neither could we detect any effects in the posterior insula that could be linked specifically to CT-touch, i.e., the CT-targeted > non-CT-targeted contrast yielded no significant results. The results of the inverse contrast were not of particular interest for our study objectives but are still included in Table 2. This contrast primarily involved areas sensitive to stimulation frequency and rhythmicity, such as the caudate nucleus, cerebellum, (pre)SMA and frontal pole^38,39^. Likewise, (pre)SMA activated during fast versus slow stimulation of the penile shaft (Fig. 3).

## Discussion

Our study reveals new insights into the affective properties of genital skin, as inferred non-invasively from the known relationship between nerve fibre response properties and psychophysical properties of touch. This approach allows for the differentiation between affective and discriminatory types of touch and has the benefit of consistent results in non-genital skin areas (primarily in the forearm)^5–11^. Essentially, this approach uses different speeds of skin brushing to target small diameter fibres that respond to innocuous mechanical stimulation. For the forearm, we confirm the ‘typical’ hedonic response to slow (CT-targeted) versus fast (non-CT-targeted) brushing stimulation. As a key finding, the psychophysical results showed that the flaccid human penis differentiates between these types of stimulation in the same way, perhaps indicating that penile skin contains nerve fibres that are optimized for detecting affective touch. However, genital stimulation overall induced stronger hedonic responses than comparable forearm stimulation.

On a brain level, the results are more ambiguous. For the forearm, we did not identify a CT-specific response in areas previously suggested to differentiate between affective versus discriminatory touch, such as S2 and pINS^7,40^. Interestingly, for the penile shaft, we did find that S2 differentiated between brushing speeds. Specifically, S2 showed a preference for the stimulation targeted at the small diameter fibres corresponding to the higher hedonic ratings. Frenulum stimulation induced the greatest reported pleasure and led to the greatest deactivation of the default-mode network. In general, the genital context of the experiment seems to have affected the neural responses, in particular to stimuli with only a mild positive valence, i.e., brushing of the forearm.

This study is the first to reveal a link between innervation of genital skin and affective responses, both at the subjective and brain level. Specifically, the results indicate that the skin of the penile shaft is likely to contain C-fibres that respond to pleasurable tactile stimulation, as found elsewhere in hairy skin^10,11,14^. It is not possible to make verifiable claims about the contribution of these fibres to sexual genital sensation as participants were asked to report general feelings of pleasure instead of sexual pleasure, while genital responses were neither recorded nor reported (with very few exceptions). While the psychophysical relationship between slow and faster brushing was similar to that in the forearm, penile stimulation evoked more pleasure than forearm stimulation. This has two possible implications that are not mutually exclusive. First, the lack of an interaction effect suggests that the human penis does not have a higher density of CT-afferents than the forearm. This raises several questions, including how relevant CT-afferents are for the strong pleasurable genital sensations typically associated with sexual behaviour. Secondly, genital stimulation overall evoked stronger pleasurable sensations than forearm stimulation, which suggests a different mechanism underlying the higher hedonic responsiveness of the penis. In this regard, the frenulum is of special interest.

Frenulum stimulation evoked the strongest pleasurable sensations of all stimulation conditions (although the difference with slow brushing of the penile shaft was non-significant). Contrary to the penile shaft, the frenulum and glans of the penis (and clitoris) are of a glabrous instead of a hairy skin type. This is noteworthy, as glabrous skin of the palms of the hands and soles of the feet is known to either lack or have a low density of CT-afferents^41–43^. Even though our results suggest that C-fibres play a role in processing pleasurable mechanical stimulation of the penile shaft, this makes it unlikely that CT-afferents specifically underlie the high pleasure potential of at least the penile frenulum and glans.

It is probable that a different type of small diameter fibre is responsible for the high affective responsiveness of the penis. For instance, the frenulum and glans are known to contain a specialized type of cutaneous receptor, the genital end bulb, that is essentially a tangled free nerve ending of a small-diameter (Aδ or C) pseudounipolar neuron^15,16^. Although it is yet unknown what quality of sensation its stimulation evokes, or to which kind of stimulation it optimally activates, it has been speculated that this type of fibre is well-suited to change shape, and probably also function, during genital vasocongestion^15,44,45^. Further research is necessary to reveal to what extent these unique genital fibres contribute to genital sensation, both inside and outside of a sexual context.

The neural responses to affective versus discriminatory forearm touch did not confirm our hypotheses. Regions that have previously been shown to discriminate between these stimulus types such as S2 and the pINS did not show a differential response to the slower versus faster brushing of the forearm. This was unexpected, as the pINS especially has previously been shown to be a key target area for CT-fibers^5–7^. For the penile shaft on the other hand, we did find a stronger S2 response to CT-targeted stimulation. This is in line with previous studies that found similar involvement of secondary somatosensory regions in the processing of CT-afferent input of the forearm and thigh regions^5,6^. Yet, similar to the forearm, the pINS did not differentiate between slower and faster brushing of the penile shaft. It should be noted that for all touch conditions we did find consistent activity in both S2 and pINS, just no significant differences between conditions, except in S2 during penile shaft stimulation. This raises the question: what could be the cause of this lack of differential activity patterns in regions assumed to process affective somatosensory information?

One possible contributing factor could be differences between the current study and others in sample sizes and analysis techniques used. Many previous studies had small sample sizes and used techniques such as region-of-interest (ROI) or multivariate analysis techniques to improve the detection of small effects^5–8^. The fact that subtle differences in statistical methodology and sample sizes can lead to different outcomes in the pINS, could raise doubts about role the pINS plays in processing affective touch. Still, the pINS has previously been shown to respond to both CT-targeted and non-CT-targeted forearm touch, although its functional coupling with other regions, including subparts of the aINS, was found to differ between conditions^40^.

The design of the present study was fundamentally different from previous studies on affective touch. Even though previous studies used similar stimulation and sometimes also multiple stimulation locations (e.g., forearm and leg), the genital context of the current experiment may have had a larger influence than we anticipated. Subtle differences in S2 and pINS activity may be masked by the strong positive hedonic responses to genital stimulation. That is, the brain might process and/or interpret afferent somatosensory signals differently in a ‘naked’ possibly even sexual context where the genitalia are exposed and even being stimulated by a partner, compared to a more neutral setting. Perhaps in such a context, the brain treats all types of somatosensory signals as potentially ‘affective’ and thus processes more of them in S2 and the posterior insula. Alternatively, the increased S2 activity might reflect enhanced attentional engagement for CT-targeted versus non-CT-targeted stimulation. S2 (and S1) activity has previously been shown to be modulated by attention, where attended vs. unattended tactile stimulation increased activity in S1 & S2^46–48^. However, the current paradigm was not intended to manipulate or measure attentional engagement, which prohibits us from drawing any firm conclusions about this. On a psychological level, participants may have been biased to give higher pleasure ratings for penile stimulation because of the generally stronger emotional connotation of genital vs. forearm stimulation. We think that this notion of ‘genital context’ effects could be an interesting topic for further study.

The penile frenulum stands out not only because of its high hedonic potential, but also because of the neural responses that its stimulation can evoke. Brushing of the penile frenulum induced the strongest deactivation of (part of) the default-mode network (DMN). If the DMN deactivation reflects task engagement, the present data suggest that this was strongest during the stimulation with the highest perceived level of pleasure. DMN deactivation in relation to affective touch has been reported before and has been suggested to be contextually dependent, with interpersonal touch capturing more attention, reflected by DMN deactivation, than impersonal touch^49^. The DMN deactivation phenomenon could thus be another reflection of the fact that CTs cannot be the sole source of, or even implicated at all, in eroticism and sexual feelings.

The main strength of the current study is the use of a paradigm that allows for a systematic analysis of psychophysical properties of touch that has proven to be reliable in many previous studies. Compared to studies that involved manual genital stimulation, the goat hair brushing stimulation at predefined speeds is more stable and the subjective and neural response that it evokes are easier to interpret. This comes at the cost of ecological validity, as goat hair brushing is a relatively neutral stimulus, compared to manual stimulation or even intercourse. Moreover, we did not deploy any measures of eroticism or (sexual) arousal, which makes it difficult to assess to what extent, if at all, the participants perceived the current experimental context as sexual. However, the few studies that have investigated eroticism in relation to CT-fibres for non-genital areas found that ratings of eroticism were highly correlated with ratings of general pleasure^50,51^. This shows how difficult it can be to disentangle general pleasure from erotic sensations, even outside of a genital context. A few participants reported having had a partial erection at some point during the paradigm, but we have no data to physiologically verify this. It would be most important for future studies to investigate the effects of vasocongestion on the current study parameters.

Perhaps the most important limitation of this study is that we only measured genital sensitivity in men. Although this was done for practical reasons—namely that the larger surface area for male genitalia allows for easier brush stimulation—we concede that this decision is reinforcing the male-biased evidence in many areas of sex research and does not address the relative paucity of data in women. Another limitation is the lack of a faster brushing control condition for the frenulum. Although the frenulum stimulation velocity was no more than 10 cm/s—which is in the optimal range for CT-fibres—there was no control condition to contrast the neural and subjective responses against. This choice was made due to the small surface area of the frenulum, which made it difficult for the partner to accurately apply faster stimulation. As a final limitation, to ensure that participants had a balanced alternation between genital and forearm stimulation, the order of conditions was pseudorandomized by the researchers and was equal for all subjects. The analysis of the psychophysical data revealed that the position of each stimulus within a stimulation block influenced reported pleasure ratings. This unintended effect of the pseudorandomization was similar for all stimulation conditions, and therefore was unlikely to have impacted the main outcomes of the study.

Even though it can be difficult to recruit for studies that involve genital stimulation, we found 19 male volunteers and their partners prepared to participate. While this may be a small sample size compared to some other neuroimaging research fields, for sexual research and even for non-sexual affective touch imaging studies this is above average. Moreover, a partnered paradigm has the benefit of reducing potential awkwardness during an intimate paradigm such as this and could also control for sexual attraction effects that have been shown to alter affective touch perception^30^. Finally, despite the lack of a control condition for the frenulum, our study is the first to assess the psychophysical properties of two different genital surface areas in a single study. Although there have been previous studies on (male) genital sensation, they tended to focus on the localization (mapping) of genitalia in S1, often by targeting only a single genital location and were not investigating the hedonic aspects of these sensations^31,34^.

This study investigated in humans the relationship between genital sensory innervation, hedonic feelings, and evoked brain responses in a systematic way. Using a tried-and-tested paradigm from affective touch research, we show that the penile shaft likely contains nerves with CT-like properties, unmyelinated small diameter nerve fibres that are tuned to a specific velocity and, when stimulated, give rise to a pleasant sensation. Still, the presence of such fibres does not fully account for the stronger overall hedonic response that we found for genital stimulation, when compared to forearm stimulation. We therefore conclude that the putative presence of CT-like fibres in the penis is insufficient for explaining the strong hedonic and erotic feelings that genital stimulation can evoke, especially in a sexual context such as during intercourse. This, combined with the higher hedonic set point for genital versus forearm touch established in the current study, leads to the conclusion that further exploration of the psychophysical properties of the genital skin is warranted. Specifically, it would be important to study how these properties might change under influence of e.g. sexual arousal and vasocongestion. On the brain level, we provide evidence that the secondary somatosensory cortex, among other areas, is implicated in pleasurable genital touch and deserves more attention in future studies. Additionally, the stimulation that evoked the greatest pleasurable sensations, frenulum stimulation, induced the greatest deactivation in (part of) the default-mode network, which could reflect the attention grasping nature of genital sensation.

## Methods

### Participants

Nineteen healthy, uncircumcised men (hereafter referred to as ‘participant’; age range 20–31 yr.; mean age 24.2 ± 3.1 yr. SD) participated in the study, together with their female partner (hereafter referred to as ‘partner’) who applied the skin stimulations. Couples were recruited through posters in university buildings and advertisements in local university and student newspapers. All participants (and partners) provided written informed consent in accordance with the Declaration of Helsinki. The study including all its procedures was approved by the internal review board of the University Medical Center Groningen, and all research was performed in accordance with relevant guidelines and/or regulations.

### Preliminary procedures and partner training

Brain responses to CT-targeted touch are known to be modulated by psychological context, including preference or non-preference of confederates applying the stimulation^30^. Our experimental set-up involved stimulation of forearm and genital skin areas, and participation of the participants' partners was assumed to make the stimulations be perceived as both natural and intimate. Couples were informed that the stimulation might elicit erection, and that if that would happen the man should not inhibit. Since the experimental set-up precluded physiological measurement of penile vasocongestion, couples were encouraged to report any indications of it. Candidate subjects were asked to indicate whether they had suffered from any psychiatric or neurological condition in the past. In addition, subjects and partners were asked to fill out the novelty seeking/harm avoidance items of the Tridimensional Personality Questionnaire^52^, as well as the standard safety checklist for magnetic resonance imaging (MRI). Subjects reporting relevant clinical conditions were excluded from participation. Subjects and partners who were within the normal range of the novelty seeking/harm avoidance subscales, and who did not meet any of the MRI exclusion criteria, were eligible for the experiment. About one week prior to the experiment, subjects eligible to participate received questionnaires regarding their sexual orientation (‘Kinsey scale’^53^), sexual self-consciousness (‘Sexual Self-Consciousness Scale’^54^), sexual function (‘International Index of Erectile Function’^55^, ‘Index of Premature Ejaculation’^56^, ‘Premature Ejaculation Diagnostic Tool’^57^], and sexual behaviour (‘Vragenlijst Seksueel Gedrag’, Dutch version of Sexual Inhibition & Sexual Excitation Scales^58^’, ’Emoties Bij Sex’ translated from Dutch as ‘Emotions In Sex’). Subjects were instructed to fill out the questionnaires without their partner present. They were subsequently invited to a sexological interview by a certified sexologist to establish any existing sexual dysfunction.

On the day of the experiment, couples arrived an hour early to prepare for the experiment and rehearse the stimulation protocol in a ‘dummy scanner’. The subject was asked to assume the supine experimental position, undressed from the waist down. The skin areas for stimulation (except the penile glans) were marked in black with a washable skin pencil, and the penis was stabilized on the lower abdomen against a small rolled-up blanket so that the left ventral surface of the shaft could be stimulated without causing too much penile movement. The prepuce was retracted over the glans. Partners were instructed as to the correct velocities, pressure, and timing at which the stimulation needed to be applied, and which body areas to stimulate. The stimulation was rehearsed until it was deemed accurate by the experimenters. After the training, but before the subject was positioned into the MRI-scanner, the subject was asked about his sexual history and the time passed since his last ejaculation before the experiment. To minimize response bias, this was done without the partner present. Finally, both subjects and partners indicated their left- or right-handedness using the ‘Edinburgh Handedness Inventory’^59^.

### Dynamic touch stimulation

**Figure 5:**
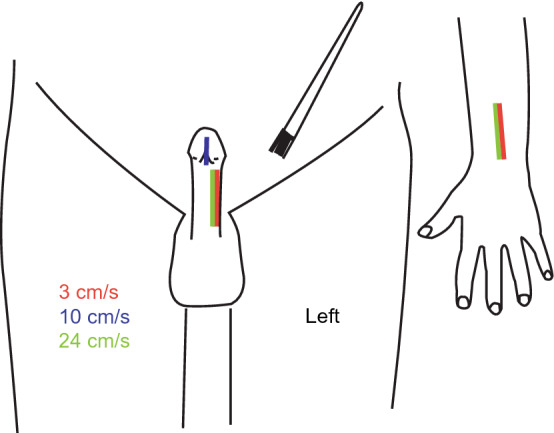
Overview of the various skin stimulation sites and corresponding velocities. The forearm and penile shaft were stimulated at velocities of approx. 3 and 24 cm/s, whereas the penile frenulum was only stimulated at approx. 10 cm/s. Figure was created using *Adobe Illustrator 2022 for Mac* (v. 26.2.1; https://www.adobe.com/products/illustrator.html).

The partner performed five different dynamic touch stimulations on the subject, distributed over three skin sites: the left forearm, the left side of the penile shaft, and the glans penis up to the frenulum (Fig. 5). The forearm was included as it is the most common stimulation site in CT-afferent research and has a dense CT innervation^11^. Stimulations of forearm and shaft were administered using a 7 cm wide soft goat-hair paint brush; glans penis stimulation was performed using a 0.5 cm wide soft make-up brush. Like the forearm, the shaft is hairy skin; glans is glabrous skin but was included because it is known as most sensitive area of the genital skin. All skin areas were stimulated in the ‘head-to-toe direction to standardize the stimulation procedure to make it easier for the partner. However, because of the most natural position of the penis in a supine body position, it inevitably meant that forearm and penis, anatomically, were brushed in opposite directions: proximal to distal for the forearm and distal to proximal for the penis.

All stimulations lasted 2 s. The timing of the brush stroking was guided by auditory cues conveyed to the partner over a headphone. First, the partner was instructed which skin area to stroke and at which velocity. Then, two low-pitched beeps were heard, followed by two high-pitched beeps, all separated by 2 s intervals. Between the high-pitched beeps the partner needed to provide the brush stroke at the indicated site, in the indicated stroke direction, and at the indicated velocity. The preceding two low-pitched beeps functioned to standardize the mechanical parameters of the partnered dynamic touch across subjects. Forearm and shaft were stimulated over a distance of 6 cm at two velocities: 3 cm/s (CT-targeted velocity) and ≥ 24 cm/s (non-CT-targeted velocity). For the CT-targeted velocity, the 6 cm distance was covered in 1 stroke at consistent speed. For the non-CT-targeted velocity, the same distance was covered in 8 strokes. The fact that there were brief intervals between brush strokes meant that the non-CT-targeted velocity was probably a bit faster than 24 cm/s. For the glans stimulation, the aim was to perform CT-targeted stimulation. However, during the experiments we learned that on the glans the stimulation distance was more variable because of inter-individual variations in prepuce retractability. Eventually, a distance between 1.5 and 5 cm—tip of the glans to (maximally) the proximal end of the frenulum—was covered 3 times in 2 s. This resulted in variable velocities that we were not reliably classifiable in relation to the CT-target range. To make the glans penis stimulations more comfortable, the partner applied a lubricant (Pjur Original) to the glans and frenulum prior to the stimulations. Thus, there were 5 dynamic touch conditions: Forearm CT-targeted; Forearm non-CT-targeted; Penile shaft CT-targeted; Penile shaft non-CT-targeted; Penile frenulum.

In addition, subjects were asked to perform two motor tasks—hand flexion and pelvic floor contraction. These tasks served to provide somatomotor references for the skin areas under investigation. The rhythm of the contractions was cued by means of an appearing and disappearing green square on the scanner screen.

### fMRI paradigm and procedure

**Figure 6:**
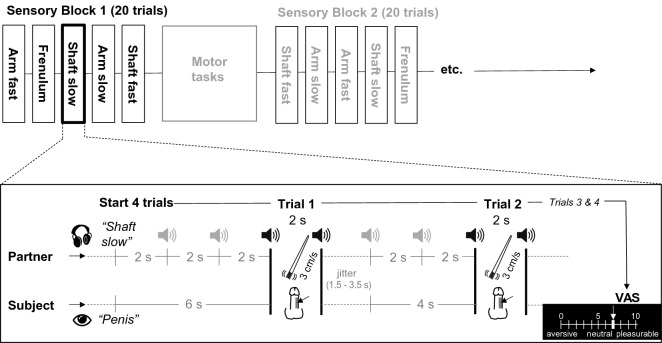
Schematic overview of the experimental task. Each sensory block consisted of 20 trials: 4 trials for each of the 5 stimulation types. Before the start of group of 4 trials, the stimulation site (forearm or penis) was presented to the subject on a screen. The partner received auditory cues indicating both the upcoming stimulation site (forearm, penile shaft, or penile frenulum) and velocity (CT-targeted or non-CT-targeted). The appropriate stimulation tempo was cued using high- and low-pitched beeps. Figure was created using *Adobe Illustrator 2022 for Mac* (v. 26.2.1; https://www.adobe.com/products/illustrator.html). Abbreviations: VAS, visual analogue scale.

The scanning session started with a resting state scan, subsequently followed by a first task session, anatomical scan and second task session. The two task sessions were identical, and each comprised three dynamic touch blocks, separated by two motor blocks. Each sensory block consisted of 20 touch trials, distributed such that there were five clusters of four trials of the same condition (Fig. 6). After each cluster, the respective dynamic touch condition was rated on pleasantness. Inter-touch intervals were jittered to reduce predictability. Thus, the experiment comprised 120 dynamic touch trials in total, and 24 trials per dynamic touch type. The order of dynamic touch conditions within each block was pseudorandomized, but the same across task sessions and participants. The somatomotor conditions went as follows. Each block comprised of five clusters of six pelvic floor or forearm muscle contractions (1.5 s contraction/1 s relaxation), yielding 30 contractions per somatomotor block. The task order within motor blocks was fixed in an alternating fashion.

### Subjective pleasantness rating

After each cluster of four brush stimulations, the subjects used a button box to rate the perceived pleasantness of the dynamic touch condition, on a scale ranging from 0 to 10; 0 meaning very unpleasant; 5 neutral and 10 very pleasant. As each condition occurred for six clusters in total, six pleasantness ratings were acquired for each type of dynamic touch per subject.

### Image acquisition

Images were acquired using a 3 T Philips Intera scanner with a sense eight-channel coil. For functional scans an echo-planar imaging (EPI) protocol with a repetition time (TR) of 2000 ms, echo time (TE) of 35 ms and 70° flip angle was used to gather whole-brain images (matrix size 64 × 64 voxels). Thirty-six slices were collected per volume in interleaved order, with an interslice gap of 0 mm, slice thickness of 3.5 mm and resulting in a 3.5 × 3.5 mm in-plane resolution. Images were acquired parallel to the anterior–posterior commissure plane. A total of 1080 (2 × 540) functional image volumes were gathered per participant. Anatomical T1-weighted images were acquired with a TR of 9 ms, TE of 3.56 ms and matrix size of 256 × 256 voxels and resting-state image volumes (300) were acquired with a TR of 2000 ms, TE of 30 ms and matrix size of 64 × 64 voxels.

Images were pre-processed and analysed using SPM12 (http://www.fil.ion.ucl.ac.uk). EPI images were realigned to a mean image of the full EPI series and subsequently the mean image was co-registered to the anatomical (T1) image. The EPI series and T1 image were then spatially normalized to MNI (Montreal Neurological Institute) standard stereotactic space. Data were resampled to 2 mm isotropic voxels. All functional volumes were smoothed with an 8 mm full-width at half-maximum isotropic Gaussian kernel. The EPI series from the resting state session were not analysed.

### Psychophysical data analysis

Psychophysical data were analysed with R (version 4.0.2; Rstudio v1.4.1106; https://rstudio.com). To assess the effects of stimulus location and velocity on subjective pleasantness scores, while controlling for individual variability, we used a linear mixed effects models (LMMs). Subsequent statistical tests on the LMM were performed using the Satterthwaite’s approximation for degrees of freedom, provided in the package lmerTest (v3.1–0; url: https://cran.r-project.org/).

For the first LMM, Pleasantness scores were entered as dependent variable, while Position within block (Early, Middle, Late), Location (Penile shaft, Forearm), Velocity (CT-targeted, non-CT-targeted), and the interaction between the latter two (Location*Velocity) were entered as (fixed) independent variables. Subject was entered as random factor (random intercept only). The subjective ratings for frenulum stimulation were not included in this interaction model, as this area was only stimulated at a single velocity which could not be reliably estimated.

For the second LMM, Pleasantness scores were again entered as dependent variable, but Position within block (Early, Middle, Late) and Stimulation condition (Forearm non-CT-targeted, Forearm CT-targeted, Penile shaft non-CT-targeted, Penile shaft CT-targeted, Frenulum) were entered as (fixed) independent variables. Subject was entered as random factor (random intercept only).

### fMRI data analysis

After pre-processing, functional brain data were analysed on 1st level (within-subject) with a fixed-effects general linear model (GLM). For each subject, the five dynamic touch and two motor conditions were modelled as boxcar functions containing the onset and duration of each trial, which were then convolved with a hemodynamic response function (HRF) and entered as variables of interest to a GLM. Rotational and translational movement parameters calculated during the realignment procedure were entered as nuisance regressors. A 128-s high-pass filter was used to remove any low-frequency drifts in the BOLD signal. We computed simple contrasts for each dynamic touch condition vs. baseline (fixation periods), for further analyses on a group (2nd) level.

We performed a whole-brain 2nd level ANOVA analysis in SPM, using the Flexible Factorial method. We specified factors ‘Subject’ (Independence: ‘Yes’; Variance: ‘Equal’) and ‘Condition’ (Independence: ‘No’; Variance: ‘Unequal). For factor ‘Condition’ we entered the individual task versus baseline contrasts for each of the dynamic touch conditions. To test the average group response per touch condition, we computed separate t-statistical maps for each. For the results of this analysis, we used a statistical threshold of at *p* < 0.05 family-wise error (FWE) corrected.

To statistically assess the effects of CT-targeted vs non-CT-targeted stimulation within the forearm and penis, we computed t-statistics for the contrasts ‘Forearm CT-optimal > Forearm CT-nonoptimal’ and ‘Penile shaft CT-optimal > Penile shaft CT-nonoptimal’ and their inverses.

The frenulum is arguably the most sensitive part of the penis with the highest pleasure potential, yet it is of a glabrous skin type. Therefore, we also wanted to explore the difference in brain responses between frenulum stimulation and CT-optimal stimulation of the penile shaft. We computed t-statistical maps for the ‘Penile shaft CT-targeted > Frenulum’ and ‘Penile shaft non-CT-targeted > Frenulum’ contrasts and their inverses.

Despite the commonly small effect sizes in affective touch research, certain brain regions such as S2 and pINS have consistently been implicated in previous studies. We therefore considered it appropriate to apply a small-volume correction (SVC) for a selected few regions-of-interest (ROIs): S1, S2, aINS, pINS, ACC. Masks for left and right S1 (BA1, BA2, BA3a, BA3b), S2 (OP1-4), aINS (Id7) and pINS (Ig1,Ig2,Id1) were defined using the JuBrain Anatomy Toolbox for SPM^60^ and a mask for the bilateral ACC was created using the Harvard–Oxford cortical structural atlas (http://www.cma.mgh.harvard.edu/fsl_atlas.html). To visualise the average response estimates for each sensory stimulation condition, we extracted the principal eigenvariates for each separate ROI in SPM (Fig. 4). We then merged all nine ROIs into a single mask which we used to apply SVC for the comparison contrasts. The FWE-corrected p-values obtained after SVC are found Table 2 denoted by an asterisk (*).

## Data Availability

All datasets used and analysed during the current study are available from the corresponding author on reasonable request.
